# Scoliose et partage - ARTBRACE

**DOI:** 10.1186/1748-7161-9-S1-O53

**Published:** 2014-12-04

**Authors:** Christine Chenot

**Affiliations:** 1 Association Scoliose et Partage, Seloncourt, France

## 

“Scoliose et Partage” is a French association of scoliosis patients. It was created in 2005 to help people suffering from scoliosis and their families. We are not a medical association.

We also want to better know and recognize scoliosis, a disease too often trivialized. We are also trying to raise awareness of the importance of early detection through a booklet created by the association and approved by the Ministry of Health.

We also collaborate when it is possible with the medical community and to this purpose we supported the doctor de Mauroy during the implementation of the ARTBRACE. For this, we analyzed psychological evaluation questionnaires (brace questionnaire) sent to young patients to see how they support and live with their new brace.

The evaluation period provides a time wearing day and night. This questionnaire includes 34 items divided into 8 groups:

1 General health perception: the majority the young people questioned are aware of the risk of development of her scoliosis

2 Physical behavior: many of them felt tired walking and had difficulty running. Sometimes it was a little harder to eat, sleep, and some breathing difficulties.

3 Emotional behavior: the ARTBRACE seems to have very little influence on the moral and character of young people.

4 Self-esteem and aesthetics: most teens do not like their physical appearance with brace

5 Vitality: they are generally more tired with the corset.

6 School activity : the brace does not seem to be a gene for school

7 Body pain: we note very little pain so drugs are rarely taken

8 Social behavior: Teenagers majority have a social life quite normal with their brace even if they sometimes had some apprehensions before.

We can conclude that the ARTBRACE is often well tolerated in all fields by young people and allow them to have a normal life.

